# Assessment of Tocilizumab (Humanized Monoclonal Antibody) for Therapeutic Efficacy and Clinical Safety in Patients with Coronavirus Disease (COVID-19)

**DOI:** 10.3390/medicina58081076

**Published:** 2022-08-10

**Authors:** Sami Ullah, Radhya Abid, Sana Haider, Fazli Khuda, Ghadeer M. Albadrani, Jawaher A. Abdulhakim, Ahmed E. Altyar, Mohamed M. Abdel-Daim, Syed Muhammad Ashhad Halimi, Atif Ali Khan Khalil

**Affiliations:** 1 Department of Pharmacy, University of Peshawar, Peshawar 25120, Pakistan; 2 Department of Pharmacy, CECOS University of Science and Technology, Peshawar 25120, Pakistan; 3 Department of Biology, College of Science, Princess Nourah bint Abdulrahman University, Riyadh 11671, Saudi Arabia; 4 Medical Laboratory Department, College of Applied Medical Sciences, Taibah University, Yanbu 46522, Saudi Arabia; 5 Department of Pharmacy Practice, Faculty of Pharmacy, King Abdulaziz University, Jeddah 21589, Saudi Arabia; 6 Department of Pharmaceutical Sciences, Pharmacy Program, Batterjee Medical College, Jeddah 21442, Saudi Arabia; 7 Department of Pharmacology, Faculty of Veterinary Medicine, Suez Canal University, Ismailia 41522, Egypt; 8 Institute of Pharmacy, Faculty of Pharmaceutical and Allied Health Sciences, Lahore College for Women University, Jail Road, Lahore 54000, Pakistan

**Keywords:** COVID-19, tocilizumab, inflammatory parameters, coagulopathy

## Abstract

*Background and objectives*: COVID-19 patients exhibit a broad range of manifestations, presenting with a flu-like respiratory tract infection that can advance to a systemic and severe disease characterized by pneumonia, pulmonary edema, severe damage to the airways, and acute respiratory distress syndrome (ARDS, causing fatality in 70% of COVID-19 cases). A ‘cytokine storm’ profile is found in most severely influenced COVID-19 patients. The treatment protocol of the disease also includes tocilizumab, which is a humanized monoclonal antibody used to treat autoimmune and inflammatory conditions. This study was designed (1) to assess the role of tocilizumab in COVID-19 patients regarding therapeutic efficacy through evaluation of cytokine release syndrome (CRS) resolution and anticoagulant effect, analyzing clinical safety via monitoring of associated adverse effects profile; and (2) to compare the clinical safety and therapeutic efficacy of institutional treatment regimen (alone) versus tocilizumab added to an institutional treatment module in COVID-19 patients. *Materials and Methods:* In this study, the endpoints parametric assessment of severely diseased patients of COVID-19 was performed (total *n* = 172, control group (institutional protocol treatment provided), *n* = 101 and test group (tocilizumab provided), *n* = 71) at the Khyber Teaching Institution, MTI, Peshawar. The assessments were compared using non-parametric analyses at baseline and after a follow-up of 12–18 days until the patient discharged or expired. *Results*: Results of the study revealed an insignificant difference among the control vs. test group in resolving inflammatory parameters (C-reactive protein (CRP) 21.30 vs. 50.07; *p* = 0.470, ferritin 482.9 vs. 211.5; *p* = 0.612, lactate dehydrogenase (LDH) 29.12 vs.18.8; *p* = 0.0863, and D-dimer 464 vs.164.4; *p* = 0.131). However, a statistically significant difference was found between the control group and test group regarding coagulation parameters (international normalized ratio (INR) 0.12 vs. −0.07; *p* ≤ 0.001; activated partial thromboplastin time (aPTT) 0.42 vs. −1.16; *p* ≤ 0.001; prothrombin time (PT) 0.31 vs. −0.96; *p* ≤ 0.001; platelet count −12.34 vs. −1.47; *p* = 0.012) and clinical survival rate (89.10 vs. 90.14; *p* < 0.001). Furthermore, there was significantly higher infection rates and raised alanine aminotransferase (ALT) and alkaline phosphatase (ALP) associated with the tocilizumab group as compared to those receiving institutional treatment (bacterial infections: 0.99% vs. 15.49%; *p* ≤ 0.01, ALT: 3.96% vs. 28.16%; *p* ≤ 0.01, ALP: 1.98% vs. 22.53%; *p* ≤ 0.01). *Conclusions*: From this study, it was concluded that tocilizumab can be a better drug of choice in terms of efficacy, particularly in resolving coagulopathy in severe COVID-19 patients.

## 1. Introduction

Severe acute respiratory syndrome coronavirus-2 (SARS-COV-2) is responsible for causing coronavirus disease-2019 (COVID-19). Patients suffering from this disease exhibit a broad range of manifestations, including a flu-like respiratory tract infection that can advance to a systemic and severe disease, which is characterized by pneumonia, pulmonary edema, severe damage to the airways, and acute respiratory distress syndrome (ARDS). ARDS is responsible to cause fatality in 70% of COVID-19 cases, in which aggressive inflammatory responses occurs advancing to septic shock, organ failure and eventually leading to death [1,2,3,4]. COVID-19 associated pneumonia is linked to high levels of inflammatory cytokines, chemokines and inflammatory mediators that are pro-inflammatory. A ‘cytokine storm’ profile is found in most severely influenced COVID-19 patients, which is distinguished by high amounts of pro-inflammatory cytokines; raised levels of interleukins (IL-18, IL-1β, IL-2, IL-6); tissue factor (TNF-α); granulocyte-colony stimulating factor (GCSF); and many chemokines (protein-10 a monocyte chemo-attractant) [5]. It is speculated that the cytokine release syndrome emulates the secondary-hemophagocytic-lymphohistiocytosis (sHLH) exhibiting a hyper-inflammatory condition, which is distinguished by a sudden and severe hyper-cytokinemia, enormous coagulation activation (leading to thromboembolic complications) and multiple organ failure [5,6,7,8,9,10,11,12].

Treatment of COVID-19 patients includes passive vaccination doses which have proved effective for pre-exposure prophylaxis of coronavirus disease. For post- exposure and symptomatic treatment, different drugs have been used worldwide on a trial and error basis, some of which are still under investigation. Based on the initial phase of the disease (a typical viral effect resulting in an increased viral disease load), antiviral treatment can be a useful option to eradicate the symptoms [6]. However, in the severe phase of the disease (a hyper-inflammatory immune response), the use of immune-modulatory treatment is more efficacious to control disease progression into the later more critical phase of COVID-19. Treatment options, i.e., remdesivir, corticosteroids (dexamethasone), monoclonal antibodies, baricitinib (Janus kinase inhibitor) and tocilizumab (IL-6 receptor antagonist), have provided promising results to eradicate its symptoms [7]. In this connection, tocilizumab has been considered as a better treatment option for managing COVID-19-induced cytokine release syndrome (CRS), because a large number of patients inflicted with severe infection manifest clinical symptoms and biochemical features redolent of CRS [6,7,8].

There are limited data reporting on the use, therapeutic efficacy and clinical safety regarding tocilizumab in severe COVID-19 patients in the Pakistan population. For these reasons, this study aimed to assess the prognostic outcomes of patients hospitalized with severe coronavirus disease and provided with institutional treatment regimen added with tocilizumab vs. those receiving institutional treatment regimens only.

## 2. Materials and Methods

### 2.1. Study Design

This open-labeled, randomized, single-center study was carried out at a tertiary care hospital (Khyber Teaching Hospital, MTI, Peshawar) on patients admitted with severe coronavirus disease, following the principles of the declaration of Helsinki and its amendments. Formal approval of the study was granted by the Ethical Research Committee of the Khyber Medical College and Teaching Hospital, MTI, Peshawar, vide testament No. 760/DME/KMC. The subjects suffering from COVID-19 were considered through positive consent, which was obtained from the patients or their next of kin. These patients were divided into two study arms:

(a) Test group of patients treated with institutional treatment protocol added with tocilizumab (the test group received tocilizumab 400 mg, IV, STAT in addition to the institutional treatment regimen). Patients were considered eligible for tocilizumab treatment if their CRP > 20 mg/dL);

(b) Reference group of patients treated with institutional treatment regimen alone (all patients received the same treatment, which included antiviral therapy (remdisivir 200 mg, IV as loading dose followed by 100 mg once daily for 4 days), antibiotic therapy (ceftriaxone 2 gm twice daily for 6 days or piperacillin/tazobactam 4.5 g, IV thrice a day or meropenem 1 gm, IV twice daily and azithromycin tablets 500 mg twice daily), anti-coagulants (prophylaxis with enoxaparin 40–60 mg subcutaneously twice a day and corticosteroid therapy (dexamethasone 1 cc, IV twice daily) [13,14].

First assessment of the set parameters was completed at baseline (before treatment), immediately after hospitalization and at days 1, 3, 7 until discharged (on average 12–18 days) or expired. All those patients were considered and evaluated who fulfilled the below-mentioned inclusion criteria [13].

Patients were included who were positively diagnosed with COVID-19 through real-time reverse-transcriptase-polymerase-chain-reaction (RT-PCR), positivity via nasopharyngeal swab, belonging to either gender and above the age of 18 years [14], severe pneumonia/respiratory involvement defined as symptoms of pneumonia presented with: respiration rate (RR) >30 breaths/min, severe respiratory distress, saturation of oxygen (SaO2) <90% while breathing ambient air or Horowitz index ≤300 mmHg [13,14], hyper-inflammatory state characterized by raised serum levels of either C-reactive protein ≥ 100 mg/L and/or ferritin ≥ 900 ng/mL along with raised lactate dehydrogenase > 220 U/L [14]. On the other hand, the major exclusion criteria included hypersensitivity to tocilizumab, concomitant bacterial infection or tuberculosis, history of diverticular disease or GI perforation, neutropenia <1500*10^9^ cells/L baseline elevated ALT/AST levels > 3–5 folds of upper limit of normal range and age <18 years [13,14].

### 2.2. Sample Size

Deploying the sample size formula for calculating the smallest size of the data set [15]:

*Minimum Sample Size = Z_1 − α/2_^2^ p (1 − p)/d^2^*

where

*Z_1 − α/2_* = is the standard normal variant (at 5% type 1 error (*p* < 0.05);

*Z* = 1.96 when confidence level (*α*) is 95%);

P = expected value within population on basis of previous research (prevalence) (i.e., 9%);

*d* = absolute error/precision (error margin)

Sample Size (*n*) ≈125.

After additionally adding up 20% of the sample for dropout probability, the final sample size was calculated to be 145. However, due to the availability of patients and to make it statistically more meaningful, a total of 172 subjects were included in the study; 101 patients were included in the institutional protocol arm (reference group) and 71 patients were in the test group.

### 2.3. Study Endpoints

The primary endpoint was the assessment of the difference/alteration in the levels of coagulation and inflammatory parameters before and after treatments provided to respective groups of patients.

### 2.4. Study Protocol

In all patients, the severity of coronavirus disease (diagnosed with RT-PCR) was measured as per the WHO’s classification, and only those patients were included in the study who were suffering from severe pneumonia, which was characterized by symptoms of pneumonia with at least one among the given: respiratory rate (RR) >30 breaths/minute, severe respiratory distress and/or saturation of oxygen < 90% on ambient air. Critical disease severity was characterized as pneumonia along with one of the given: (a) acute respiratory distress syndrome (ARDS); (b) sepsis; or (c) septic shock [13,14,16]. The assessments of both groups were carried out at baseline, days 1, 3, and 7 after initiation of treatment and follow-up until discharged (on average 12–18 days) or expired.

#### 2.4.1. Demographics, Biochemical and Hematological Assessment

Assessments were made based on recorded demographic and required information (age, gender, socioeconomic status), co-morbidities (smoking history, chronic kidney disease (CKD), cancer, diabetes mellitus, arterial hypertension (HTN), chronic obstructive pulmonary disease (COPD) and coronary artery disease (CAD)), chief complaints on hospital admission (sign and symptoms), duration of symptoms, thrombosis risk determinants (e.g., cancer, prior venous thromboembolism (VTE), cardiovascular disease (CVD) [13,14]), patients’ biochemical and hematological data (liver function tests (LFTs), acute neutrophil count (ANC), blood coagulation profile: routine clotting assays including international normalized ratio (INR), prothrombin time (PT), activated partial thromboplastin time (aPTT), plasma levels of D-dimer, serum platelet count), inflammatory parameters (ferritin, lactate dehydrogenase (LDH), C-reactive protein (CRP)); patients’ clinical data respiratory parameters: FiO_2_, peripheral oxygen saturation, the Horowitz index for lung function viz. PaO_2_/FiO_2_ ratio, need for supplemental oxygen, non-invasive ventilation (NIV) or invasive ventilation requirement, and body temperature.

#### 2.4.2. Clinical Assessment

The status of each patient was clinically evaluated using a six-category ordinal scale for clinical improvement and survival, which was specified as: 1 = patient discharged or meeting discharge criteria (defined as clinical recovery and symptomatic relief); 2 = hospitalization without supplemental oxygen; 3 = hospitalization with supplemental low-flow oxygen, i.e., FiO_2_ < 40%); 4 = hospitalization needing high-flow supplemental oxygen (FiO_2_ ≥ 40%) and/or non-invasive ventilation; 5 = hospitalization requiring invasive mechanical ventilation or ECMO (Extra-Corporeal Membrane Oxygenation); 6 = death/expiry. In general, the overall clinical improvement and survival was defined as discharge from the hospital and/or reduction of a minimum of 2 points on the six-category ordinal scale in comparison to baseline value [16,17,18]; patients’ radiological data (Chest X-Rays (CXR) and CT scans); monitoring patients’ clinical safety (tocilizumab-related common adverse events, *viz*. injection site related hypersensitivity reactions, elevation of alanine aminotransferase (ALT/SGPT), development of bacterial infection/sepsis or fungal infection such as candidemia and pulmonary aspergillosis (immune-suppression associated opportunistic infections), neutropenia (by delaying IL-6 regulated release of neutrophils from the bone marrow), and gastrointestinal perforations (via IL-6 inhibition, which shows gastro-protective activity by preventing epithelial apoptosis during prolonged inflammation) [13,14,19,20].

### 2.5. Data Analysis

The statistical analysis of data was carried out using SPSS version 21. Percentages (%) mean (average), and frequency (*n*) together with standard deviation and standard error of mean were calculated using descriptive statistics. Distribution of data and normality was calculated for parametric or non-parametric assessment. The correlation and association between numerical and categorical variables was conducted and intergroup and intragroup comparison was carried out utilizing appropriate tests: namely, *t*-test or Mann–Whitney/Wilcoxon test; ANOVA, Fisher’s Exact or Kruskal–Wallis H test (as needed). The probability value, i.e., *p*-value for statistically significant results was calibrated at *p* < 0.05.

## 3. Results

This study was conducted from March 2020 until August 2021, where 172 positively diagnosed cases of severe COVID-19 were treated with either institutional protocol treatment (control group, *n* = 101) or with tocilizumab added with institutional regimen (test group, *n* = 71). Included patients were assessed at baseline and follow-up until expiry or discharged. Analysis of clinical safety and therapeutic efficacy among the treatment arms was carried out on the basis of set parameters.

### 3.1. Patients’ Demographics and Clinical Presentations

Results obtained from the study showed a significant difference regarding gender variability among treatment groups with *p* = 0.004. However, the ages of the participants included in both groups were comparable and insignificantly different (*p* = 0.194). Co-morbidities viz. diabetes mellitus (*p* = 0.762), hypertension (*p* = 0.49), chronic obstructive pulmonary disease (*p* = 0.074), and smoking (*p* = 0.688) were statistically insignificantly different between two treatment groups, except cancer (*p* = 0.049) and chronic kidney disease (*p* = 0.003), which were significantly different between the two arms. Risk factors for thrombosis, i.e., cardiovascular disease (*p* = 0.095) and history of thrombo-embolism (*p* = 0.402) were statistically insignificantly different between two treatment arms. Clinical symptoms: fever (*p* = 0.649), cough (*p* = 0.762), shortness of breath (*p* = 0.091), generalized body pain (*p* = 0.207), chest pain (*p* = 0.373), loss of appetite (*p* = 0.402) and diarrhea (*p* = 0.457) were also reported as comparable between the said treatment groups on the basis of their *p*-values. The duration of hospitalization (*p* = 0.192), duration of symptoms (*p* = 0.42) and fatal cases (*p* = 0.41) were also found with a statistically insignificant difference. A further detailed description is given in Table 1.

### 3.2. Assessment of Clinical Safety

Parametric assessment revealed that there was an insignificant difference in the clinical survival rate between the two treatment groups (89.10% vs. 90.14%). Conversely, the percentages of reported bacterial infections and raised ALT and ALP cases in the tocilizumab arm are higher as compared to the control group, showing the adverse drug events and relatively lower safety profile of tocilizumab regarding the said indicative items (i.e., bacterial infections: 0.99% vs. 15.49%; ALT: 3.96% vs. 28.16%; and ALP: 1.98% vs. 22.53%). Further details are listed in Table 2.

### 3.3. Assessment of Therapeutic Efficacy

Group-to-group comparison was performed to assess therapeutic efficacy of the treatment regimens under investigation. Among the inflammatory parameters, there was an insignificant difference regarding the lowering of CRP between the treatment arms with *p* = 0.470, ferritin (*p* = 0.612) and LDH (*p* = 0.0863). Similarly, regarding the coagulation parameters, an insignificant difference was recorded in the lowering of D-dimer (*p* = 0.131). However, the differences in the INR, aPTT and PT between the control group and test group were highly significant, i.e., *p* < 0.001. These results showed that the tocilizumab-treated group of the patients had better mitigated coagulation parameters than those of the control group. Furthermore, the difference between the platelet counts of both the groups was also statistically significant, but the mean difference was lower in the control group than the test group, showing that institutional protocol treatment reduced the platelet count more than the tocilizumab group. Similarly, the difference of clinical survival rate between the two groups was also statistically significant. Further details are given in Table 3.

### 3.4. Therapeutic Efficacy from Baseline to Endpoint

The therapeutic efficacy of treatment regimens was assessed considering the *p*-values of the investigated parameters at baseline and follow-up, such that CRP (*p* < 0.001), LDH (*p* < 0.001), ferritin (*p* < 0.001), INR (*p* = 0.024), aPTT (*p* = 0.003), PT (*p* = 0.036), and platelets (*p* < 0.001) showed highly significant difference. Nevertheless, the D-dimer (*p* = 0.691) was insignificantly different.

**Figure 1:**
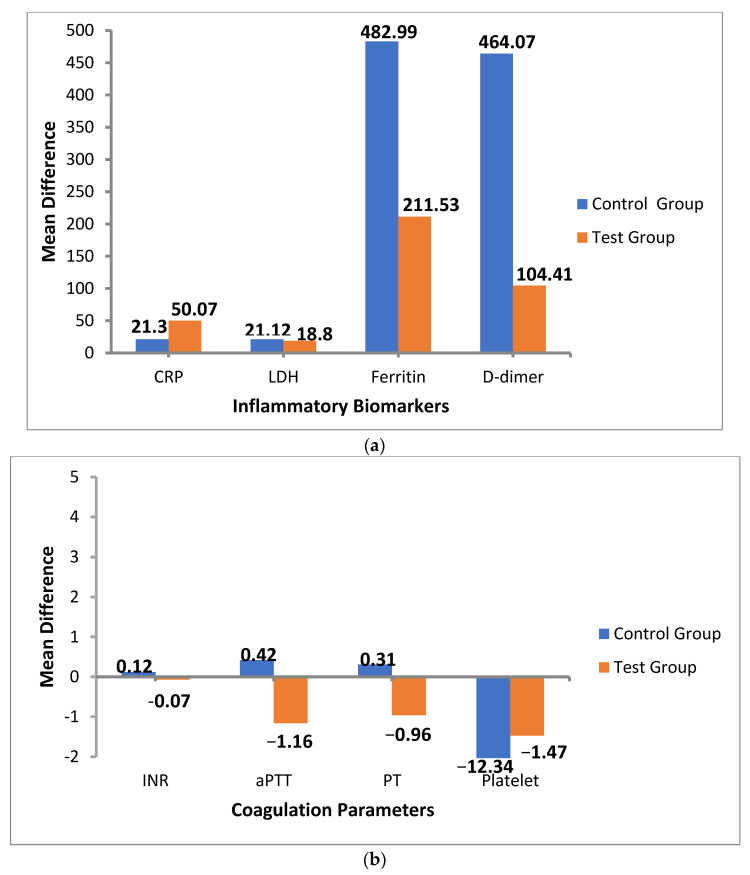
(**a**) Therapeutic efficacy determined (in both test group (*n* = 71) and control group (*n* = 101)) by considering the difference of the values of the inflammatory biomarkers assessed at baseline and follow-up intervals. (**b**) Therapeutic efficacy determined (in both test group (*n* = 71) and control group (*n* = 101)) by considering the difference of the values of the coagulation parameters assessed at baseline and follow-up intervals.

Similarly, an assessment of parameters, i.e., CRP (*p* < 0.001), D-dimer (*p* < 0.003), ferritin (*p* < 0.001), INR (*p* = 0.024), aPTT (*p* = 0.003), PT (*p* = 0.036), and platelets (*p* < 0.004) at baseline vs. follow-up in the test arm (tocilizumab treated added with institutional regimen) were found to have a statistically highly significant difference except in LDH (*p* < 0.114), which was insignificant. Further details are explained in Table 4 and Figure 1a,b.

## 4. Discussion

Generally, the findings of the study have revealed that the differences of mean among the inflammatory parameters and coagulation parameters in the tocilizumab and institutional protocol groups were found to be lower in comparison to the only institutional protocol group, except for CRP and platelet count, which were higher in the tocilizumab arm, showing that tocilizumab effectively mitigates all inflammatory and coagulation parameters except for CRP and platelet count, which were better mitigated in the test group receiving only institutional protocol (CRP 21.30 (12.2) vs. 50.07 (10.43), ferritin 482.9 (116.7) vs. 211.5 (52.26), LDH 29.12 (10.18) vs. 18.8 (13.9), D-dimer 464 (193.8) vs. 164.4 (71), INR 0.12 (0.08) vs. −0.07 (0.01), aPTT 0.42 (0.31) vs. −1.16 (0.19), PT 0.31 (0.24) vs. −0.96 (0.11), and platelet count −12.34 (6.3) vs. −1.47 (5)).

Studies have reported that coronavirus-associated pneumonia is linked to high levels of inflammatory cytokines, chemokines and inflammatory mediators that are pro-inflammation. A ‘cytokine storm’ profile is found in most severely influenced COVID-19 patients known as ‘cytokine release syndrome’ (CRS), which is a hyper-immune and hyper-inflammatory response distinguished by raised levels of cytokines, tissue necrosis factor (TNF-α), interleukins (IL-6, IL-2, IL-18, IL-1β); and many chemokines. These elevated pro-inflammatory mediators are responsible for causing lung damage by raising the level of C-reactive protein (CRP) from liver via IL-6 transduction and activation. Moreover, studies have reported that there is a formation of neutrophil extracellular traps (NET) and monocyte recruitment within the lung micro-vessels in COVID-19 patients with severe disease causing leakage due to endothelial cell dysfunction, leading to pulmonary edema and disseminated intravascular coagulation manifested by raised levels of D-dimers, lactate dehydrogenase (LDH), ferritin and increased duration of prothrombin time (PT), activated partial thromboplastin time (aPTT), international normalized ratio (INR) and raised levels of platelet count [6,7,8,9,10,11].

For COVID-19 patients, many different treatment options, unless they show any contraindication, are considered including monoclonal antibodies for SARS-COV-2 (e.g., etesevimab, imdevimab), antiviral drugs (e.g., remdesivir, ritonavir, lopinavir), anti-inflammatory medications (e.g., dexamethasone), and immune-modulatory drugs (e.g., baricitinib, tocilizumab); these are being researched and evaluated for the possible treatment and management of COVID-19 [21]. Interleukin-6 is believed to play a pivotal role in the cytokine release syndrome and systemic inflammation and coagulation in severe COVID-19 patients and IL-6 receptor blockade by immune-modulatory agent; i.e., tocilizumab has gained popularity in the treatment of COVID-19 [14]. This drug not only inhibits the IL-6 receptor but also inhibits Factor-XIII, which is an important clotting factor in the coagulation cascade, therefore resolving the systemic inflammation as well as coagulopathy. According to research by Corrado Campochiaro, among the COVID-19 patients that were initially given tocilizumab, 69% of them improved clinically during the 28-day follow-up period in comparison to 61% of patients treated with standard treatment. Mortality in the tocilizumab group was also lower, i.e., 15% compared to 33% in the standard treatment group [14,22].

Similar to our findings, as per the research study conducted by Salvatore et al., the tocilizumab-treated group showed worsened liver function tests particularly for ALT in comparison to those not receiving tocilizumab [23]. In this relation, a research study conducted by Kok Hoe Chan and others reported that 18 among 24 patients, i.e., 75% who received tocilizumab showed an elevated level of D-dimer, which was similar to our findings. Furthermore, a reduction in levels of CRP, ferritin and LDH was reported in tocilizumab-treated patients [24]. Another research conducted by Marcello Di Nisio et al. found that COVID-19 patients that were treated with tocilizumab reported an improvement in elevated D-dimer levels, reduced levels of CRP, prothrombin time and aPTT, as with our outcomes. However, an increase in the levels of platelets was reported, which was contrary to our findings [13]. Another study by Corrado Campochiaro et al., as with our data, concluded that tocilizumab-treated patients showed an increased occurrence of bacterial infections compared to those who did not receive tocilizumab [25]. In accordance to our study findings, research by Campochiaro et al. found out that the clinical symptoms of most of the patients resolved after receiving the treatment; 49% subjects of the standard care versus 63% in the test group treated with tocilizumab were discharged, having a mean time of discharge at approximately 13.5 days (2 weeks) after treatment with tocilizumab compared to 14 days for patients treated with standard care [15]. Similarly, as with our findings, in another study by Xiaoling et al., the average hospitalization time of patients was recorded to be 15 days after the administration of tocilizumab [26]. Furthermore, in research carried out by Giovanni Guaraldi et al., tocilizumab in comparison to standard care alone was reported to be a less safe option: patients treated with tocilizumab developed more secondary infections compared to those treated with only standard care, i.e., 24/179 (13%) in the tocilizumab group vs. only 14/365 (4%) in the standard care group, which was similar to the results obtained by our research study [27].

Similar to our outcomes is a research study conducted by Stokes et al. elucidating that among 0.375 million symptomatic COVID-19 confirmed cases in America, approximately 70% of patients presented with fever, cough, and shortness of breath, around 36% of cases experienced myalgia, and almost 34% of cases reportedly had a headache [28]. In addition, in accordance with our study, analysis carried out by Mukherjee et al. revealed that the severity of COVID-19 was more in males compared to females [29]. Similarly, studies conducted by Karyono and Wicanksana, Wei-Jie Guan et al., Irawaty Djaharuddin et al., and clinical data released by The American College of Cardiology reported that a higher mortality rate was found in COVID-19 patients suffering from diabetes, hypertension and cardiovascular diseases [30,31,32].

## 5. Conclusions

It was concluded that tocilizumab (added with institutional treatment module) showed better therapeutic efficacy than institutional treatment protocol (alone) in terms of resolving inflammation (by lowering inflammatory parameters except for CRP), coagulopathy (reducing coagulation parameters except platelet count) and cytokine release syndrome (CRS), thereby improving disease prognosis in COVID-19 patients.

## 6. Future Recommendations

The futuristic study design will become more beneficial if conducted at multi-centers and considering the relatively large sample size of the patients suffering from COVID-19 treated by tocilizumab. It will also be a better option to exclude all those patients suffering from bacterial co-infections. Furthermore, considering the pharmacoeconomics aspect of the treatment arms can also provide sufficient information to decide the better treatment option.

## Figures and Tables

**Table 1:** Demographics and clinical characteristics of the COVID-19 patients provided with respective treatment module in control vs. test group (*n* = 172).

Variables	Control Group *n* = 101 (58.72%)	Test Group *n* = 71 (41.27%)	*p*-Value
Demographics:			
Gender			0.004
Male	55 (54.45)	54 (76.05)	
Female	46 (45.54)	17 (23.94)	
Age, mean ± S.D (range)	61.01 ± 12.3 (40–110)	62.154 ± 8.81 (42–77)	0.194
Co-Morbidities:			
T2DM	55 (54.45)	37 (52.11)	0.762
HTN	58 (57.42)	37 (52.11)	0.492
COPD	22 (21.78)	8 (11.26)	0.074
CKD	12 (11.88)	0	0.003
Smoking	4 (3.96)	2 (1.98)	0.688
Cancer	11 (10.89)	2 (1.98)	0.049
CAD	28 (27.72)	13 (18.30)	0.327
Risk Factors for Thrombosis:			
Cancer	11 (10.89)	2 (1.98)	0.049
History of thromboembolism	1 (0.99)	0	0.402
CVD	52 (51.4)	45 (63.38)	0.095
Symptoms:			
Fever	42 (41.58)	21 (29.57)	0.649
Cough	55 (54.45)	37 (52.11)	0.762
Shortness of breath	101 (100)	48 (67.60)	0.091
Generalized body pain	54 (53.46)	31 (43.66)	0.207
Chest pain	4 (3.96)	5 (7.04)	0.373
Loss of appetite	1 (0.99)	0	0.402
Diarrhea	7 (6.93)	3 (4.22)	0.457
Duration of hospitalization	12.613 ± 3.072 (9–28)	12.95 ± 2.52 (9–22)	0.192
Duration of symptoms	11.91 ± 3.184 (8–28)	11.4647 ± 2.45 (7–20)	0.42
Fatal Cases	9(8.9%)	7(9.8%)	0.797

T2DM, Type-2 diabetes mellitus; HTN, Hypertension; COPD, Chronic obstructive pulmonary dis ease; CKD, Chronic kidney disease; CAD, Coronary artery disease; CVD, Cardiovascular disease. Values expressed *n* (%).

**Table 2:** Prevalence of treatment associated adverse drug effects/complications to compare clinical safety of test and control treatment groups, *n* = 172.

Variables	Control Group *n* = 101	Treatment Group *n* = 71	*p*-Value
Bacterial Infections, *n* (%)	1 (0.99)	11 (15.49)	<0.001
Raised ALT, *n* (%)	4 (3.96)	20 (28.16)	<0.001
Raised ALP, *n* (%)	2 (1.98)	16 (22.53)	<0.001

ALT, Alanine aminotransferase; ALP, Alkaline phosphatase. Values expressed *n*/N (%) and *p*-value.

**Table 3:** Parametric assessment to compare therapeutic efficacy in control vs. test group (*n* = 172).

Variables	Control Group *n* = 101	Test Group *n* = 71	*p*-Value
CRP	21.30 ± 12.2	50.07 ± 10.43	0.470
Ferritin	482.9 ± 116.7	211.5 ± 52.26	0.612
LDH	29.12 ± 10.18	18.8 ± 13.9	0.0863
INR	0.12 ±0.08	−0.07 ± 0.01	<0.001
Aptt	0.42 ± 0.31	−1.16 ± 0.19	<0.001
PT	0.31 ± 0.24	−0.96 ± 0.11	<0.001
Platelets	−12.34 ± 6.3	−1.47 ± 5	0.012
D-dimer	464 ± 193.8	164.4 ± 71	0.131
Clinical survival rate	90 (89%)	64 (91%)	<0.05

PT, Prothrombin time; CRP, C-reactive protein; aPTT, Activated partial thromboplastin time; LDH, Lactate dehydrogenase; INR, International normalized ratio. Expressed values; mean, standard error (SE), *n* (%) and *p*-values.

**Table 4:** Comparison of inflammatory and coagulation parameters from baseline to follow-up in treatment groups (*n* = 172).

Variables	Control Group *n* = 101			Test Group *n* = 71		
	Baseline	Follow-Up	*p*-Value	Baseline	Follow-Up	*p*-Value
CRP	111.76 ± 9.43	90.45 ± 12	<0.001	105.8 ± 12.7	55.76 ± 5.78	<0.001
Ferritin	1378.7 ± 120.7	895.7 ± 53.27	<0.001	1131.7 ± 54.7	926.1 ± 60.8	<0.001
LDH	353.5 ± 15.28	324.3.1 ± 15	<0.001	330.7 ± 10.2	311.8 ± 16.3	0.114
INR	1.16 ± 0.08	1.04 ± 0.01	0.024	0.98 ± 0.01	1.06 ± 0.01	<0.001
aPTT	65.35 ± 0.38	64.93 ± 0.313	0.003	63.60 ± 0.18	64.77 ± 0.20	<0.001
PT	12.8 ± 0.24	12.48 ± 0.12	0.036	12.25 ± 0.19	13.21 ± 0.15	<0.001
Platelets	210.9 ± 10	223.32 ± 9.27	<0.001	181.7 ± 9.43	183.1 ± 5.89	0.004
D-dimer	2541.5 ± 307.7	2077 ± 218	0.691	842.28 ± 131.7	737.8 ± 135.4	0.003

PT, Prothrombin time; CRP, C-reactive protein; aPTT, Activated partial thromboplastin time; LDH, Lactate dehydrogenase; INR, International normalized ratio. Expressed values; mean, standard error (SE), *n* (%) and *p*-value.

## Data Availability

Not applicable.
